# Switching antifibrotics in patients with idiopathic pulmonary fibrosis: a multi-center retrospective cohort study

**DOI:** 10.1186/s12890-021-01587-3

**Published:** 2021-07-12

**Authors:** Yuzo Suzuki, Kazutaka Mori, Yuya Aono, Masato Kono, Hirotsugu Hasegawa, Koshi Yokomura, Hyogo Naoi, Hironao Hozumi, Masato Karayama, Kazuki Furuhashi, Noriyuki Enomoto, Tomoyuki Fujisawa, Yutaro Nakamura, Naoki Inui, Hidenori Nakamura, Takafumi Suda

**Affiliations:** 1 Second Division, Department of Internal Medicine, Hamamatsu University School of Medicine, 1-20-1 Handayama Higashi-ku, Hamamatsu, Shizuoka 431-3192 Japan; 2 Department of Respiratory Medicine, Shizuoka City Shimizu Hospital, Hamamatsu, Japan; 3 Department of Respiratory Medicine, Seirei Hamamatsu General Hospital, Hamamatsu, Japan; 4 Department of Respiratory Medicine, Seirei Mikatahara General Hospital, Hamamatsu, Japan

**Keywords:** Antifibrotic therapy, Idiopathic pulmonary fibrosis, Switch of antifibrotics

## Abstract

**Background:**

Currently, there are two antifibrotics used to treat idiopathic pulmonary fibrosis (IPF): pirfenidone and nintedanib. Antifibrotics slow disease progression by reducing the annual decline of forced vital capacity (FVC), which possibly improves outcomes in IPF patients. During treatment, patients occasionally switch antifibrotic treatments. However, prognostic implication of changing antifibrotics has not yet been evaluated.

**Methods:**

This multi-center retrospective cohort study examined 262 consecutive IPF patients who received antifibrotic therapy. Antifibrotic agents were switched in 37 patients (14.1%). The prognoses were compared between the patient cohort that switched antifibrotics (Switch-IPF) and those without (Non-Switch-IPF) using propensity-score matched analyses.

**Results:**

The median period between the initiation of antifibrotic therapy and the drug switch was 25.8 (12.7–35.3) months. The most common reasons for the switch were disease progression (n = 17) followed by gastrointestinal disorders (n = 12). Of the 37 patients that switched antifibrotics, only eight patients disrupted switched antifibrotics by their adverse reactions. The overall prognosis of the Switch-IPF cohort was significantly better than the Non-Switch-IPF cohort (median periods: 67.2 vs. 27.1 months, *p* < 0.0001). In propensity-score matched analyses that were adjusted to age, sex, FVC (%), history of acute exacerbation, and usage of long-term oxygen therapy, the Switch-IPF cohort had significantly longer survival times than the Non-Switch-IPF group (median 67.2 vs. 41.3 months, *p* = 0.0219). The second-line antifibrotic therapy showed similar survival probabilities than those in first-line antifibrotic therapy in multistate model analyses.

**Conclusion:**

Switching antifibrotics is feasible and may improve prognosis in patients with IPF. A further prospective study will be required to confirm clinical implication of switching the antifibrotics.

**Supplementary Information:**

The online version contains supplementary material available at 10.1186/s12890-021-01587-3.

## Take home message

Switching antifibrotics were generally well-tolerated. The prognosis of IPF patients switching antifibrotics was significantly better than those not switching, suggesting that switching is feasible and may improve prognosis in patients with IPF.

## Introduction

Idiopathic pulmonary fibrosis (IPF) is a progressive fibrosing form of interstitial lung disease (ILD) with unknown aetiology [1–3]. IPF is characterized by progressive cough and dyspnea together with decreased pulmonary function, which eventually leads to respiratory failure. Two antifibrotics, pirfenidone and nintedanib, are currently used to treat IPF in a clinical setting. These antifibrotics have, with similar efficacies, been shown to reduce the annual decline of forced vital capacity (FVC) in patients with IPF [4–7]. Additionally, nintedanib has been shown to slow FVC decline in patients with systemic sclerosis-associated ILD [8] and other types of progressive fibrosing ILDs [9]. Unfortunately, respiratory function is not improved or stabilized with either of these drugs.

These two antifibrotics have distinct types of antifibrotic mechanisms [10] and different adverse drug reaction (ADR) profiles [11–15]. In the clinical setting, the choice of either pirfenidone or nintedanib is likely to be made partially in view of their profiles of ADRs. In real-world practice, switching antifibrotics is occasionally performed due to ADRs or progressive diseases. If the first antifibrotics is or becomes ineffective, the second one may provide benefit because of its different antifibrotic mechanism. This is the case for the treatment of lung cancer. However, the efficacy and feasibility of switching antifibrotics have not been assessed yet. Furthermore, because only two drugs are proven to be effective for IPF at present, we must use them ingeniously. This study aimed to evaluate the prognostic implications and feasibility of switching antifibrotics in patients with IPF.

## Methods

### Subjects

This retrospective study was conducted on 312 consecutive patients with ILD, receiving pirfenidone or nintedanib, at Hamamatsu University of School of Medicine, Seirei Hamamatsu Hospital, and Seirei Mikatahara Hospital. All patients were treated between February 2009 and March 2020. Fifty ILD patients were excluded from the study: 37 patients were diagnosed with non-IPF ILD, and 13 IPF patients had insufficient clinical data. Thus, this study enrolled 262 patients with IPF. The IPF patients switching antifibrotics (Switch-IPF) was defined as patients who received first-line antifibrotics more than one month and followed by second-line antifibrotic therapy. The IPF patients who received first-line antifibrotics less than one months or were not treated with second-line antifibrotics were defined as Non-Switch-IPF. Diagnosis of IPF was based on the ATS/ERS/Japanese Respiratory Society (JRS)/Latin American Thoracic Association (ALAT) criteria [1–3]. The study protocol was approved by the Ethical Committee of Hamamatsu University School of Medicine (17–196) and carried out in accordance with approved guidelines. The need for patient approval and/or informed consent was waived due to the retrospective nature of the study, and was approved by the Ethical Committee of Hamamatsu University School of Medicine.

### Data collection

Clinical data were obtained from the patients’ medical records. Laboratory findings and pulmonary and functional test results obtained at the time of starting antifibrotic therapy were recorded. Acute exacerbation (AE) was diagnosed based on the ATS guidelines [16, 17]. The cases with disease progression that met the following criteria in comparison to the initiation of antifibrotic therapy were included: a relative decline in the FVC of at least 10% of the predicted value; a relative decline in the FVC of 5% to less than 10% of the predicted value and worsening of respiratory symptoms or an increased extent of fibrosis on high-resolution CT; worsening of respiratory symptoms and an increased extent of fibrosis on high-resolution CT [9].

### Statistical analysis

Discrete variables were expressed as totals (percentages), and continuous variables were expressed as the median [interquartile range]. The Mann–Whitney U test was used to compare the continuous variables. Fisher’s Exact test for independence was used to compare categorical variables. Overall survival time was measured from the start date of first-line antifibrotic therapy, unless otherwise specified. Propensity-matched analyses were employed to determine the impact of switching the antifibrotics on prognosis. Propensity score matching was performed using the following algorithm: 1:1 optional match with a ± 0.05 calliper and no replacement. Univariate and multivariate analyses were also performed using the Cox proportional hazards regression model. The variables of propensity score matching and multivariate Cox-regression analyses that were considered clinically important, and specific risk factors for mortality in IPF were selected [2, 3, 15, 18–20].

As “switching antifibrotics” was a time-varying event, the values of “switching antifibrotics” was evaluated using time-dependent Cox regression analysis and Markov multistate model to describe the clinical transitions of second-line antifibrotic therapy [21–23]. Cumulative survival probabilities from initiation of antifibrotic therapy were calculated using the Kaplan–Meier method and the Log-Rank test. An event was defined as death from any causes and patients who lost or still alive were censored at the last follow-up date.

As “switching antifibrotics” was a time-varying event, we evaluated the values of “switching antifibrotics” by time-dependent Cox regression analysis and Markov multistate model to describe the clinical transitions of second-line antifibrotic therapy. The Markov multistate models have traditionally been used to study effect of transitory state of illness (or interventions such as transplantation) on prognoses to death [21–23]. The transient states were defined as following; individuals initiated first-line antifibrotic therapy (state 1), those initiated second-line antifibrotic therapy (state 2), and death (state 3). The transition time from state 1 to state 2 was defined as the difference between date of initiation of first-line antifibrotic therapy and start date of second-line antifibrotic therapy. Similarly, death (transition to state 3) was taken as the date from the last entry into either state 1 or state 2 to date of death. From the Markov model estimates for both transition intensities and probabilities from one state to another were obtained. The former summarizes the instantaneous risk of transition between any two states and is analogous to a hazard rate whereas the latter is an estimate of the probability of transitioning to a different state or time [22].

Statistical analyses were performed using GraphPad Prism Version 6 (GraphPad Software, San Diego, CA, USA), JMP (Ver13, SAS Institute, Inc., Cary.NC, USA), and R software version 4.0.2 (The R Foundation for Statistical Computing, Vienna Austria). All analyses were two-tailed and *p* values < 0.05 were considered statistically significant.

## Results

### Clinical characteristics

The clinical characteristics at the initiations of first-line antifibrotics of IPF patients with Switch-IPF) and Non-Switch-IPF are shown in Table 1. Among the 262 patients with IPF, 37 patients (14.1%) switched antifibrotics. Of these, 29 patients switched from pirfenidone to nintedanib, whereas the remaining patients switched from nintedanib to pirfenidone. The history of acute exacerbation was significantly higher in patients with Non-switch IPF. Both cohorts comprised male IPF patients who were around 70 years old and were regular smokers. Antifibrotics were administered at median 14.1 months after IPF diagnosis. Because pirfenidone was approved before nintedanib, it was more frequently given as the first-line antifibrotic than nintedanib among the Switch-IPF cohort. Pulmonary function tests showed that the Switch-IPF cohort tended to have preserved FVC (%) compared with the Non-Switch-IPF cohort. A lower frequency of long-term oxygen therapy (LTOT) usage was found in the Switch-IPF cohort compared to the Non-Switch-IPF cohort.

**Table 1:** Clinical characteristics of 262 patients with IPF treated with antifibrotic therapy

	Switch-IPF cohort (n = 37)	Non-Switch-IPF cohort (n = 225)	Non-Switch-IPF Continued cases (n = 177)	Non-Switch-IPF Discontinued cases (n = 48)	Non-Switch IPF versus Switch-IPF *p* value
Age, years	70.0 [65.5–74.0]	73.0 [68.0–77.0]	72.0 [67.5–76.0]	74.0 [68.0–79.0]	0.0616
Sex, male/female	31 (83.8%)/6 (16.2%)	185 (82.2%)/40 (17.8%)	147 (83.0%)/30 (17.0%)	38 (79.2%)/10 (20.8%)	1.000
cIPF/UIP/IPF	31 (83.8%)/6 (16.2%)	175 (77.8%)/50 (22.2%)	134 (75.7%)/43 (24.3%)	41 (85.4%)/7 (14.6%)	0.5186
Diagnosis to antifibrotic therapy, months	13.6 [1.7–31.5]	15.4 [2.8–47.2]	12.1 [2.6–44.1]	31.5 [5.6–66.1]	0.2324
Pirfenidone/Nintedanib	29 (78.4%), 8 (21.6%)	130 (57.8%), 95 (42.2%)	102 (57.6%)/75 (42.4%)	28 (58.3%)/ 20 (41.7%)	0.0184
History of AE	0 (0%)	33 (14.7%)	25 (14.1%)	8 (16.7%)	0.0067
Never/former & current smoker	6 (16.2%), 31 (83.8%)	46 (20.4%), 179 (79.6%)	37 (20.9%)/140 (79.1%)	9 (18.8%)/39 (81.3%)	0.6602
Smoking pack-year	40.0 [18.0–59.0]	30.0 [3.0–46.0]	30.8 [3.0–45.0]	30.0 [7.5–48.0]	0.1297
BMI, kg/m^2^	24.3 [21.3–25.3]	22.9 [20.7–25.4]	23.3 [21.4–25.7]	21.9 [19.5–23.5]	0.2329
*Pulmonary function test*					
FVC, %-pred	73.2 [62.4–83.7]	67.7 [56.5–79.8]	68.3 [58.5–80.2]	61.4 [47.7–77.4]	0.1018
FEV_1_, %-pred	75.3 [67.6–87.6]	74.4 [63.3–92.2]	76.4 [64.5–90.9]	73.1[55.8–90.9]	0.7480
FEV_1_/FVC, %	83.4 [79.9–88.9]	86.4 [79.9–92.2]	85.4 [79.8–91.4]	90.2 [80.4–94.5]	0.0438
DLCO, %	62.2 [50.2–67.6] (n = 37)	58.3 [43.4–72.0] (n = 185) Unable to perform (n = 14)	58.4 [44.0–71.5] (n = 153) Unable to perform (n = 7)	57.6 [38.1–77.7] (n = 32) Unable to perform (n = 7)	0.3516
GAP stage, I, II, III	18 (48.6%), 19 (51.4%), 0 (0%)	76 (38.2%), 90 (45.2%), 33 (16.6%)	63 (39.4%), 74 (46.3%), 23 (14.4%)	13 (33.3%), 16 (41.0%), 10 (25.6%)	0.0267
*6-min walk test*					
Distances, m	432 [345–515] (n = 28)	400 [310–484] (n = 134)	400 [308–484] (n = 116)	400 [180–482] (n = 18)	0.1990
Minimum SpO_2_ < 90%	19/28 (67.9%)	100/134 (74.6%)	87/116 (75.0%)	13/18 (72.2%)	0.4845
*UCG*					
TRV ≥ 2.9 m/s	2 (8.7%) (n = 29)	24 (17.9%) (n = 134)	16 (15.4%) (n = 104)	8 (26.7%) (n = 30)	0.1719
*Laboratory*					
Hb, g/dl	14.0 [13.2–15.1]	13.5 [12.3–14.7]	13.6 [12.4–14.8]	13.3 [12.1–14.7]	0.0612
TP, g/dl	7.5 [7.2–7.9]	7.4 [6.9–7.8]	7.5 [7.0–7.8]	7.3 [6.7–7.8]	0.0855
Alb, g/dl	4.1 [4.0–4.2]	3.9 [3.6–4.1]	3.9 [3.6–4.1]	3.7 [3.5–4.0]	0.0006
LDH, U/L	241 [210–270]	230 [203–273]	228 [203–268]	244 [206–280]	0.8415
CRP, mg/dl	0.2 [0.1–0.5]	0.3 [0.1–0.6]	0.3 [0.1–0.6]	0.3 [0.1–0.6]	0.5156
KL-6, U/ml	1124 [776–1473]	1062 [798–1524]	1057 [796–1560]	1105 [817–1402]	0.7003
SP-D, ng/ml	232 [136–345]	249 [165–370]	247 [158–375]	251 [188–369]	0.7278
*Treatment*					
None	30 (81.1%)	128 (56.9%)	126 (71.2%)	22 (45.8%)	0.0060
LTOT	6 (16.2%)	85 (37.8%)	65 (36.7%)	20 (41.7%)	0.0144
*Flow rate during rest*					
< 1, 1–3, > 3, L/min	4, 2, 0	40, 42, 3	30, 32, 3	10, 10, 0	
Immunosuppressants	3 (8.1%)	50 (22.2%)	37 (20.9%)	13 (27.1%)	0.0486
PSL	3	30	23	8	
PSL + CyA	0	11	7	4	
PSL + CPA		5	3	2	
PSL + Tac		4	4	0	

*AE* acute exacerbation, *BMI* body mass index, *FVC* forced vital capacity, *FEV*_*1.0*_ forced expiratory volume in 1.0 s, *DLCO* diffuse capacity of the lung for carbon monoxide, *GAP* Gender–Age–Physiology, *UCG* ultrasound echocardiogram, *TRV* Tricuspid regurgitant jet velocity, *KL-6* Krebs von den Lunge-6, *SP-D* surfactant protein-D, *LTOT* long-term oxygen therapy, *PSL* prednisolone, *CyA* cyclosporine A, *CPA* cyclophosphamide, *Tac* tacrolimus

Among Non-Switch-IPF cohort, 48 patients (21.3%) discontinued the antifibrotics during the observation period with a median exposure time of 7.2 months. The characteritics of Non-Switch-IPF Continued cases and Discontinued cases were also presented in Table 1. The most common reason for discontinuation was gastrointestinal (GI) side effects including nausea, appetite loss, and diarrhoea (n = 25, 52.1%), followed by liver enzyme elevation (n = 6, 12.5%, Additional file 3: Table S1). The remaining patients continued to take the antifibrotics for the entire duration of the study.

### Causes of switching antifibrotics and its timing

Among the Switch-IPF patients, the most common cause of switching the antifibrotics was disease progression (PD, n = 17), followed by GI side effects (n = 12) (Table 2). Furthermore, photosensitivity and elevated liver enzymes were also observed in two patients.

**Table 2:** Causes for switching antifibrotics in first-line treatment and causes for discontinuation of second-line antifibrotics

First-line treatment (n = 37) [Pirfenidone (n = 29), Nintedanib (n = 8)]		Second-line treatment (n = 37) Nintedanib (n = 29), Pirfenidone (n = 8)	
Disease progression	17 (45.9%), [2, 15]	Gastrointestinal side effects	4 (5.4%), [1, 3]
Gastrointestinal disorders	12 (32.4%), [3, 9]	Rash	1 (2.7%), [1, 0]
Photosensitivity	2 (5.4%), [2, 0]	Rash and dizziness	1 (2.7%), [0, 1]
Liver enzyme elevation	2 (5.4%), [1, 1]	Dizziness	1 (2.7%), [1, 0]
Peripheral eosinophilia	1 (2.7%), [1, 0]	Gastrointestinal perforation	1 (2.7%), [1, 0]
Lung cancer development	1 (2.7%), [1, 0]		
Vasospastic angina suspected	1 (2.7%), [0, 1]		
Patients’ will	1 (2.7%), [1, 0]		

**Fig. 1:**
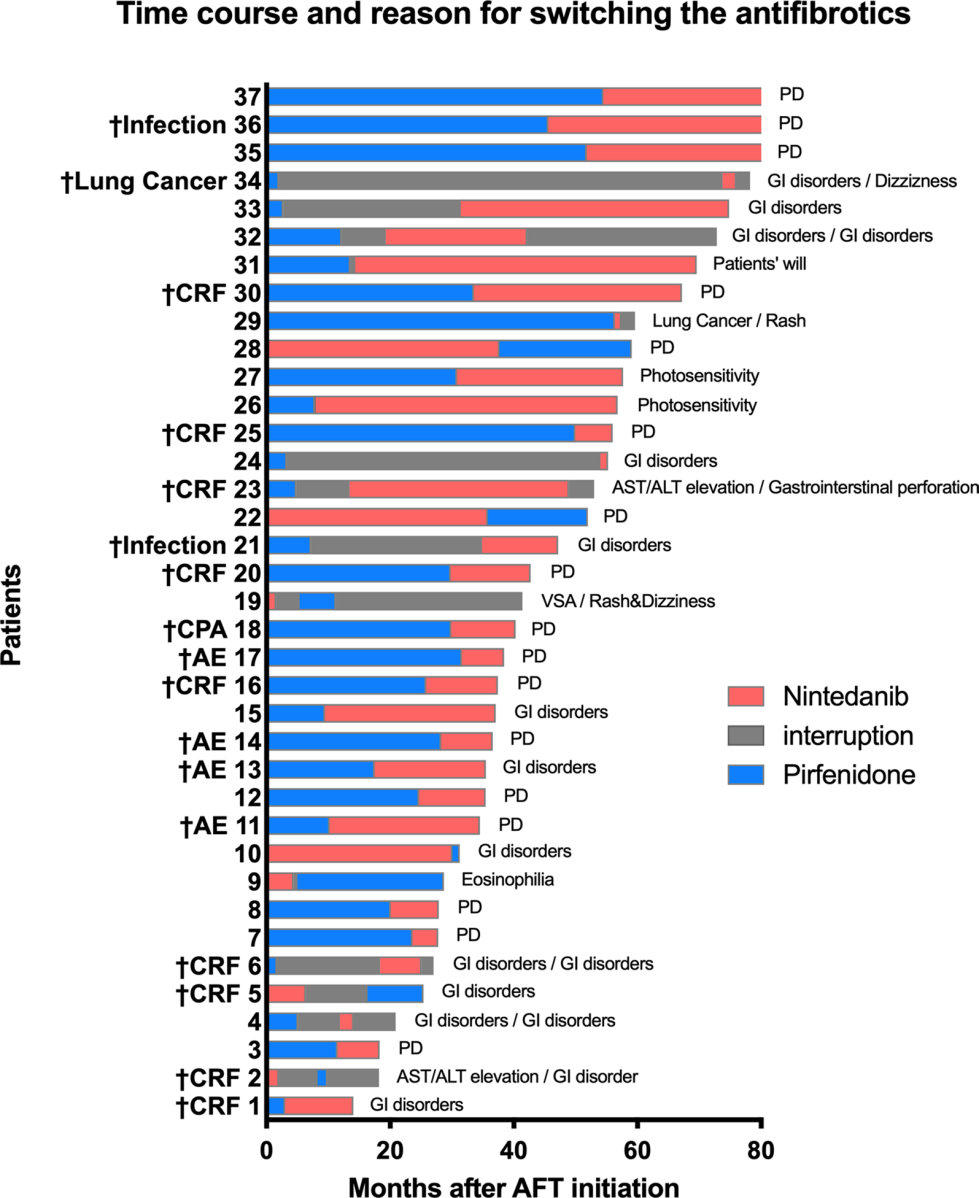
Time course and reason for switching the antifibrotics. Time course and reasons for switching the antifibrotics in each patient. *CRF* chronic respiratory failure, *AE* acute exacerbation, *CPA* cardiac arrest

The detailed time course and reasons for switching antifibrotics are shown in Fig. 1 and Additional file 3: Table S2. The exposure period to first-line antifibrotics and the interval from the end of first-line treatment to the initiation of second-line treatment were 17.5 (5.0–31.2) months and 0 (0–7.0) months, respectively. Patients who switched antifibrotics due to PD had a significantly longer exposure time to the first-line antifibrotics compared to patients who switched due to reasons other than PD [29.9 (24.2–41.6) months vs. 5.7 (2.8–13.2) months, respectively, *p* < 0.0001]. Patients who switched due to PD also had significantly shorter intervals from the end of first-line treatment to the initiation of second-line treatment compared to the patients who switched for other reasons [0 (0–0) months vs. 5.3 (0–15.8) months, respectively, *p* < 0.0001]. Patients who switched antifibrotics due to Non-PD, mostly due to adverse drug reactions (ADRs), were initiated second-line treatment delayed from after resolutions of ADRs.

### Pulmonary function tests between initiations of first-line antifibrotic therapy and initiations of second-line antifibrotic therapy

**Fig. 2:**
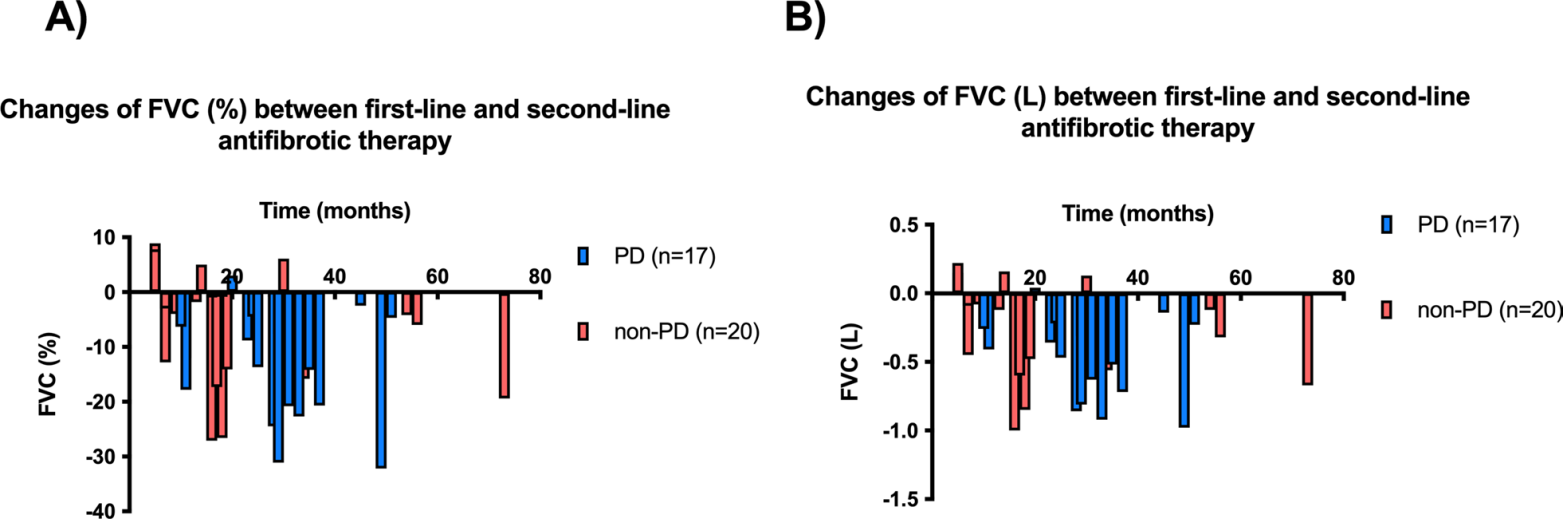
Changes of FVC (%) and FVC (L) between initiations of first-line antifibrotic therapy and initiations of second-line antifibrotic therapy. Changes in FVC (%) (**A**) and FVC (L) (**B**) between initiations of first-line and those of second-line antifibrotic therapy. X axis represents months between initiations of first-line and those of second-line antifibrotic therapy. Y axis shows relative (**A**) and absolute (**B**) declines of FVC between initiations of first-line and those of second-line antifibrotic therapy. Each bar depicts a single patient. Two patients were not examined spirometry at initiations of second-line antifibrotic therapy

The declines of FVC (%) and FVC (L) between the first and second-line treatments are shown in Fig. 2. At the initiation of second-line antifibrotics, the FVC (%) and FVC (L) in Switch-IPF patients dropped 8.8 (4.0–19.4) % and 0.32 (0.12–0.63) L, respectively, from the initiation of first-line antifibrotics. Although the decline in FVC (%) and FVC (L) was greater among patients that switched antifibrotics due to PD than among those that switched due to other reasons [14.0 (5.1–22.2) % vs. 6.5 (1.8–15.8) %, and 0.44 (0.24–0.79) L vs. 0.27 (0.08–0.56) L, respectively], the difference was not statistically significant.

### Feasibility of second-line antifibrotics

The clinical characteristics at the initiations of second-line antifibrotics were showed in Additional file 3: Table S3. Second-line antifibrotics were tolerable in most cases (82.2%). However, eight patients discontinued second-line antifibrotics due to ADRs (Fig. 1; Table 2). The most common ADR associated with the discontinuation was GI side effects. All patients who switched due to PD had good tolerability to second-line antifibrotics, while eight out of 20 patients that switched antifibrotics due to other reasons did not.

### Causes of death and prognosis

**Fig. 3:**
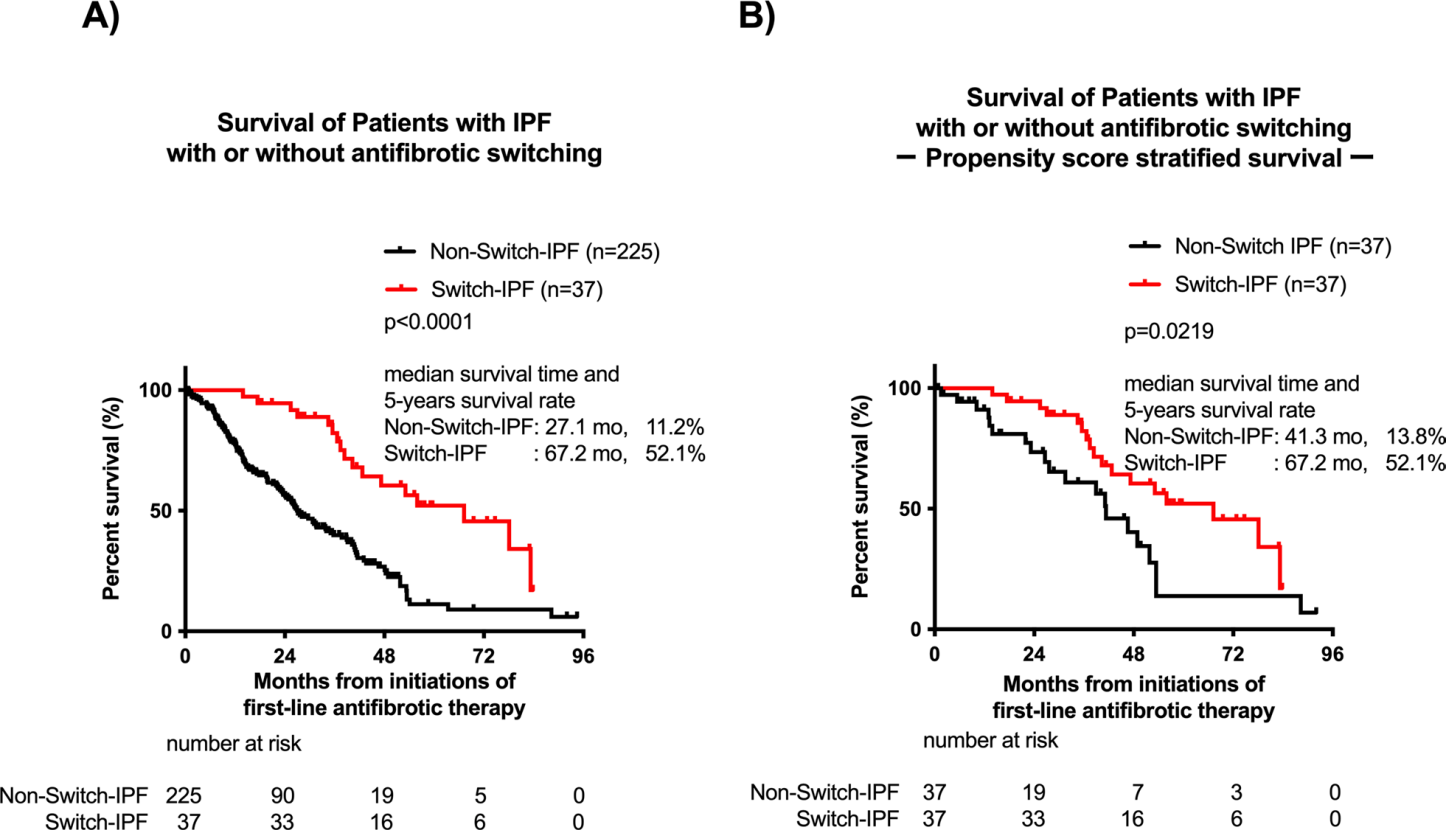
Survivals of patients with IPF with or without switching antifibrotics: Switched cases versus Non-switched cases. Survival of patients with IPF with or without antifibrotic switching (**A**). Survival of propensity-matched patients with or without antifibrotic switching (**B**). *p* values were determined by the log-rank test. Cumulative survival probabilities from initiation of antifibrotic therapy were calculated. An event was defined as death from any cause and patients who were lost or still alive were censored at the last follow-up date

During the observation period, 17 patients in the Switch-IPF cohort and 130 patients in the Non-Switch-IPF cohort died. The most common cause of death was chronic respiratory failure, followed by AE. The causes of death did not differ between the Switch-IPF and Non- Switch-IPF patients (Table 3). However, the median survival time and 5-year survival rate of Switch-IPF patients were significantly longer than that of the Non-Switch-IPF patients (median survival time: 67.2 vs. 27.1 months; 5-year survival rate: 52.1% vs. 11.2%, respectively) (Fig. 3A). When stratified according to Gender–Age–Physiology (GAP) system, patients with the Switch-IPF showed longer survival than those with the Non-Switch-IPF patients in both GAP stage I and GAP stage II (Additional file 1: Fig. S1). Among the Switch-IPF cohort, no prognostic differences were observed for antifibrotics or causes of switching (Additional file 2: Fig. S2).

**Table 3:** Cause of mortality in patients with IPF treated with antifibrotic therapy

	Switch-IPF (n = 17)	Non-Switch-IPF All cases (n = 130)	Non-Switch-IPF Continued cases (n = 96)	Non-Switch-IPF Discontinued cases (n = 34)	Non-Switch IPF versus Switch-IPF *p* value
Chronic respiratory failure	9 (52.9%)	74 (56.9%)	55 (57.3%)	19 (55.9%)	0.7988
Acute exacerbation	4 (23.5%)	32 (24.6%)	23 (24.0%)	9 (26.5%)	1.0000
Lung cancer	1 (5.9%)	6 (4.6%)	5 (5.2%)	1 (2.9%)	0.5850
Pneumothorax	0 (0%)	6 (4.6%)	6 (6.3%)	0 (0%)	1.0000
Infection	2 (11.8%)	3 (2.3%)	3 (3.1%)	0 (0%)	0.1024
Others	1 (5.9%)	9 (6.9%)	4 (4.2%)	5 (14.7%)	1.0000

As shown in Table 1, the clinical backgrounds of Switch-IPF and Non- Switch-IPF patients differed, specifically in terms of FVC (%), AE history and LTOT usage. Thus, we adjusted the patients’ age, sex, FVC (%), AE history, and LTOT usages at initiation of first-line antifibrotic therapy using propensity-matched analyses. Thirty-seven well-matched pairs were extracted, and their clinical characteristics are shown in Additional file 3: Table S4. No significant differences were found between the matched pairs at initiations of first-line antifibrotic therapy, including the duration of antifibrotic treatment. However, after adjustments, Switch-IPF patients still exhibited significantly better prognoses than Non- Switch-IPF patients (*p* = 0.0219, Fig. 3B).

**Fig. 4:**
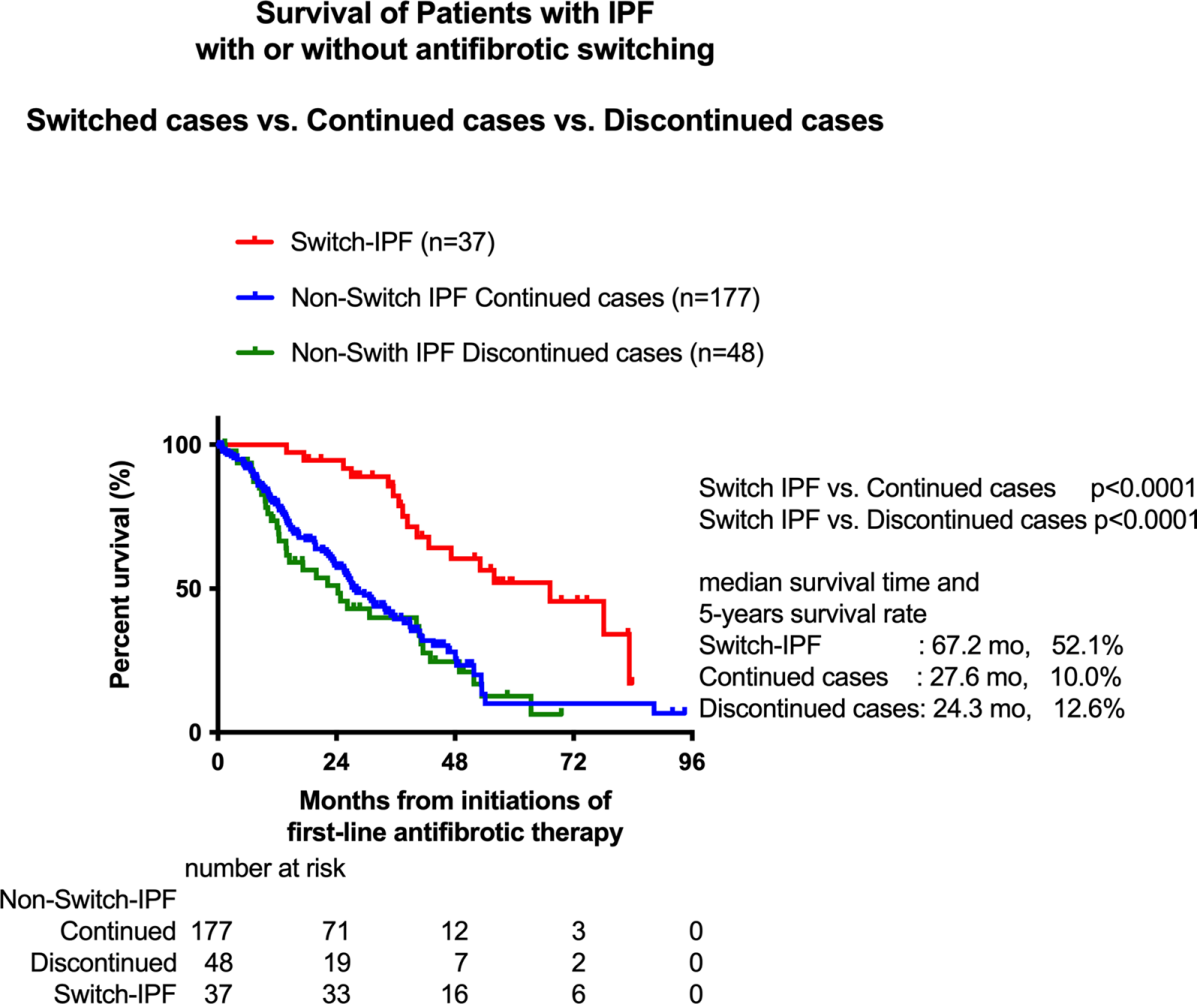
Survivals of patients with IPF with or without switching antifibrotic: Switched cases versus Non-Switch IPF Continued cases versus Non-Switch IPF Discontinued cases. Survival of patients with IPF with or without antifibrotic switching. Switch-IPF versus Non-switch-IPF Continued cases versus Non-switch-IPF Discontinued cases. *P* values were determined by the log-rank test. Cumulative survival probabilities from initiation of antifibrotic therapy were calculated. An event was defined as death from any cause and patients who were lost or still alive were censored at the last follow-up date

Not all Non-Switch-IPF patients received antifibrotics for the entire duration of the study; 48 patients (21.3%) discontinued antifibrotic treatment during the observation period, whereas the rest patients (n = 177) were continued first-line antifibrotic therapy. We therefore compared the survival times of Switch-IPF patients with Non-Switch-IPF Discontinued cases and Non-Switch-IPF Continued cases, respectively. The Switch-IPF patients still exhibited significantly longer survival times than Non-Switch-IPF Discontinued cases or Non-Switch-IPF Continued cases (Fig. 4).

### Differences in prognosis calculated from diagnosis of IPF

**Fig. 5:**
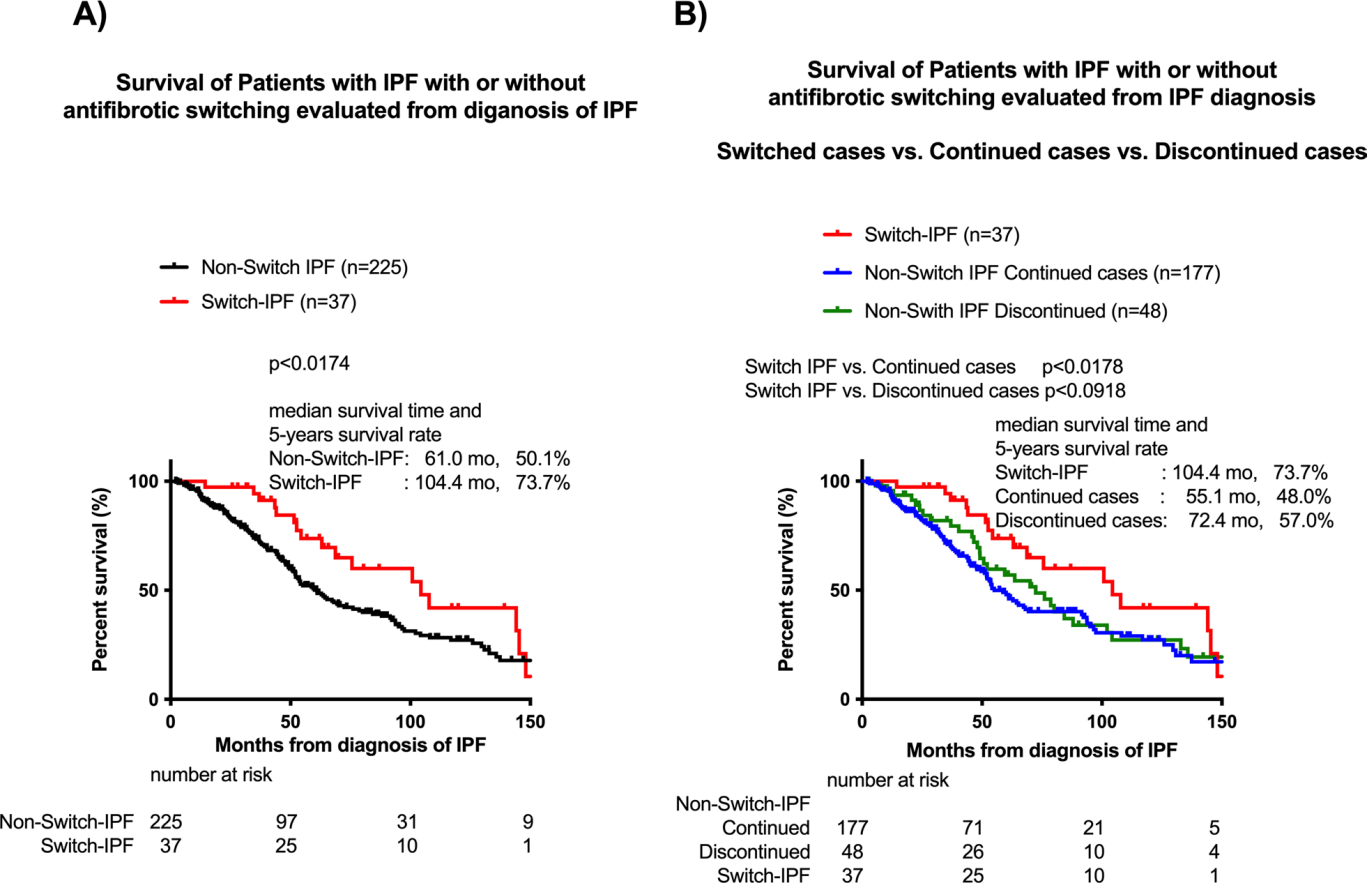
Survival of patients with IPF with or without switching antifibrotics, as evaluated from diagnosis of IPF. Survival of patients with IPF with or without antifibrotic switching evaluated from diagnosis of IPF (**A**). Switch-IPF versus Non-switch-IPF Continued cases versus Non-switch-IPF Discontinued cases (**B**). *P* values were determined by the log-rank test. Cumulative survival probabilities from diagnosis of IPF were calculated. An event was defined as death from any cause and patients who were lost or still alive were censored at the last follow-up date

The prognoses from the diagnosis of IPF were also evaluated in patients in the Switch-IPF, the Non-Switch-IPF, the Non-Switch-IPF Discontinued cases, and the Non-Switch-IPF Continued cases. Regardless of the timing of IPF diagnosis or initiation of antifibrotic therapy, patients with Switch-IPF showed better prognoses than the Non-Switch-IPF patients (Fig. 5A). Although the prognostic difference was not statistically significant (vs. Discontinued cases), patients in the Switch-IPF cohort consistently showed better prognoses (Fig. 5B).

### Prognostic implications of “switching antifibrotics” and prognosis of “patients with IPF who switched antifibrotics”

As “switching antifibrotics” was a time-varying event, we evaluated the values of “switching antifibrotics” by time-dependent Cox proportional analysis. The time-dependent Cox proportional analysis showed hazard ratio of “switching antifibrotics” were less than 1.0 but not significant (Table 4). Whereas Cox proportional univariate analyses and multivariate analyses adjusted by age, sex, history of AE, FVC (%), and LTOT usages revealed that “patients with IPF who switched antifibrotics” were identified as a population with better prognoses (Table 4).

**Table 4:** Prognostic implications of switches of the antifibrotics in patients with IPF by univariate and multivariate Cox-proportion analyses

Predictor	HR	95% CI	*p* value		HR	95% CI	*p* value
*Univariate analysis*				*Multivariate analysis 1*			
Age, year	1.024	1.000–1.050	0.0481	Age, year	1.003	0.978–1.029	0.8402
Gender, male	1.054	0.652–1.624	0.8218	Gender, male	1.455	0.905–2.438	0.1365
History of AE, yes	2.413	1.518–3.677	< 0.0001	History of AE, yes	1.062	0.602–1.777	0.8268
Pirfenidone	1.002	0.694–1.425	0.9892	BMI, per 1 kg/m^2^ increase	0.956	0.904–1.009	0.1051
Period: Diagnosis-administration	1.001	0.996–1.005	0.7124	FVC, per 1% increase	0.978	0.967–0.989	0.0001
BMI, per 1 kg/m^2^ increase	0.905	0.860–0.953	0.0001	LTOT, yes	1.774	1.205–2.590	0.0032
FVC,per 1% increase	0.970	0.959–0.980	< 0.0001	Patients with IPF who switched antifibrotics	0.392	0.221–0.656	0.0007
FEV_1_, per 1% increase	0.989	0.979–0.999	0.0261	*Multivariate analysis 2*			
FEV_1_/FVC, per 1% increase	1.061	1.038–1.089	< 0.0001	Age, year	1.005	0.979–1.032	0.7131
DLCO, per 1% increase	0.974	0.962–0.986	< 0.0001	Gender, male	1.536	0.940–2.618	0.0992
TP, per 1 g/dl increase	0.978	0.763–1.271	0.8647	History of AE, yes	1.214	0.684–2.267	0.5247
Alb, per 1 g/dl increase	0.601	0.411–0.896	0.0105	BMI, per 1 kg/m^2^ increase	0.940	0.883–0.997	0.0440
KL-6, per 1 U/ml increase	1.000	1.000–1.000	0.0003	FVC, per 1% increase	0.980	0.968–0.991	0.0007
SP-D, per 1 ng/ml increase	1.001	1.000–1.002	0.0510	KL-6, per 1 U/ml increase	1.000	1.000–1.000	0.0217
LTOT, yes	2.575	1.847–3.574	< 0.0001	LTOT, yes	1.660	1.103–2.468	0.0135
Patients with IPF who switched antifibrotics	0.318	0.182–0.506	< 0.0001	Patients with IPF who switched antifibrotics s	0.374	0.206–0.638	0.0006
Switching antifibrotics (time dependent covariate)	0.895	0.517–1.550	0.692				

*AE* acute exacerbation, *BMI* body mass index, *FVC* forced vital capacity, *FEV*_*1.0*_ forced expiratory volume in 1.0 s, *DLCO* diffuse capacity of the lung for carbon monoxide, *LTOT* long-term oxygen therapy

### Efficacies of second-line antifibrotic therapy

**Fig. 6:**
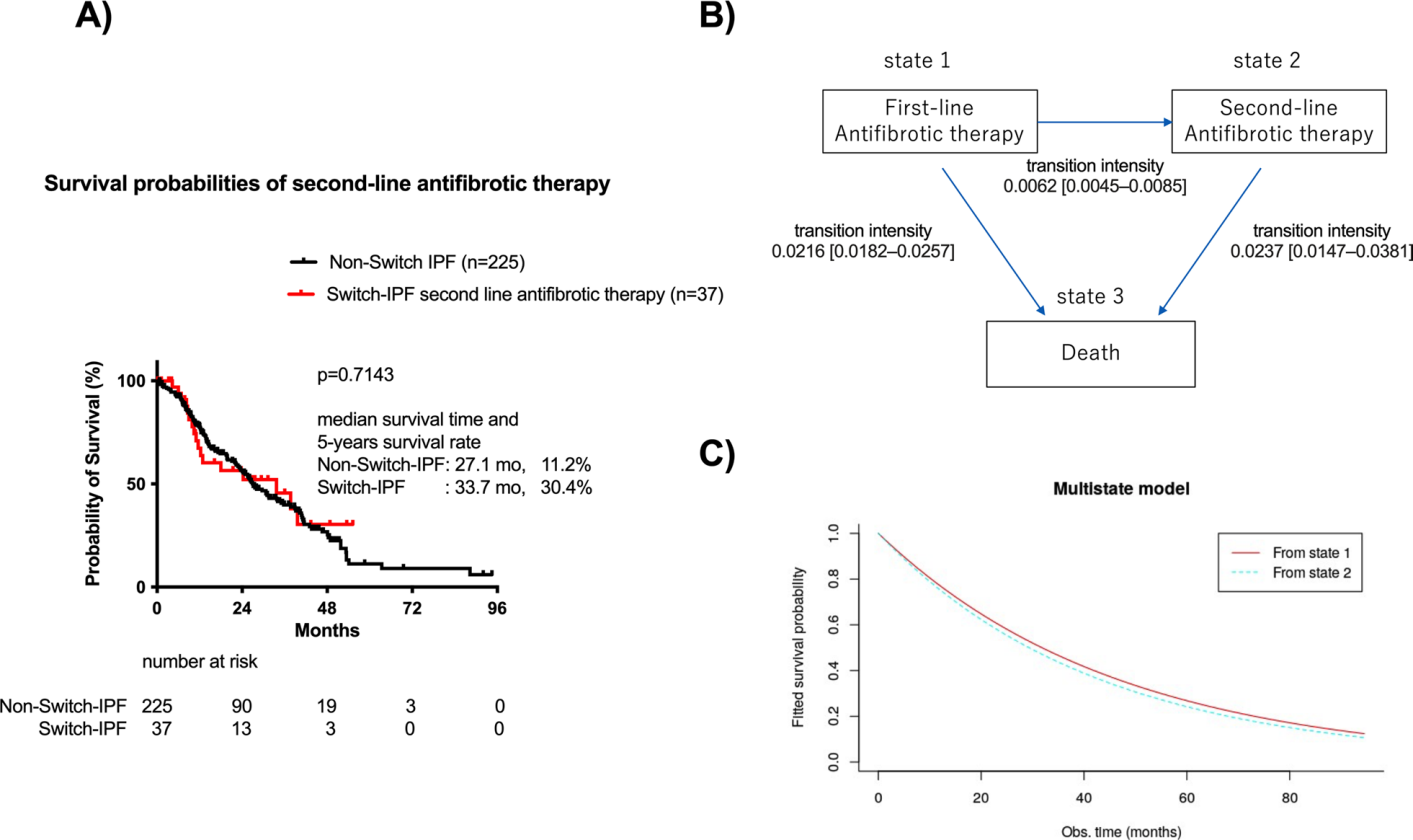
Fitted survival probabilities of first-line and second-line antifibrotic therapy. The cumulative survival probabilities from initiation of second-line AFT in patients with Switch-IPF and survival probabilities of first-line AFT in patients with Non-Switch-IPF (**A**). Diagram showing the multistate model used for modeling the impact of “switching antifibrotic therapy” on survival in patients with IPF (**B**). Fitted survival probability curves based on transition intensities from state 1 to state 3 (i.e., transitioning to death without switching antifibrotics) and state 2 to 3 (i.e., transitioning to death after switching antifibrotics) in the unadjusted multistate model (**C**)

To justify immortal-time bias and to evaluate efficacies of second-line antifibrotic therapy, survival probabilities of second-line antifibrotic therapy in patients with Switch-IPF were evaluated using the Kaplan–Meier method and the Markov multistate model. The cumulative survival probabilities from initiation of second-line antifibrotic therapy, in patients with Switch-IPF were similar to those of first-line antifibrotic therapy in patients with Non-Switch-IPF (Fig. 6A). The Markov multistate model was aimed to describe the clinical transitions of second-line antifibrotic therapy. In the Markov multistate models, the transient states of individuals are presented in Fig. 6B. The results of the unadjusted Markov multistate model summarizing transition intensities between states are presented; From state1, individuals had a positive risk of transitioning to state2 [transition intensity = 0.0062 (0.0045–0.0085)], and death [transition intensity = 0.0216 (0.0182–0.0257)]. Meanwhile, from state2 individuals had similar transitioning risk for death [transition intensity = 0.0237 (0.0147–0.0381)] compared with those from state1. Subsequently, IPF patients that initiated second-line antifibrotic therapy presented similar survival probabilities than those in first-line antifibrotic therapy (Fig. 6C).

## Discussion

The present study for the first time examined feasibility and prognostic implications of switching antifibrotics in patients with IPF. Among 262 IPF patients receiving antifibrotics, 37 patients were subjected to switching antifibrotics from one to another. The most common cause for the switch was disease progression. Of the 37 patients, only 8 patients disrupted 2nd line antifibrotic treatment due to ADRs. Overall, IPF patients switching antifibrotics (Switch-IPF) had a significantly longer survival than those not (Non-Switch-IPF), and this survival benefit remained significant in propensity-matched analysis adjusted by age, sex, FVC (%), AE history and LTOT usages. Further, second-line antifibrotic therapy showed similar survival probabilities than those in first-line antifibrotic therapy using a multistate model analyses. These results suggested that the switch of antifibrotics is feasible and may have a prognostic benefit in patients with IPF.

Currently, the antifibrotic medications, pirfenidone and nintedanib, are available for the treatment of IPF. Nintedanib inhibits multiple tyrosine kinases, including the receptors for vascular endothelial growth factor, fibroblast growth factor, and platelet-derived growth factor. The underlying mechanism of action for pirfenidone is still unknown [10], but it is different from that of nintedanib. Thus, the combined administration of these two antifibrotics may be more effective than the administration of a single antifibrotic. In this context, a recent Phase II clinical study demonstrated that nintedanib, with add-on pirfenidone, had manageable safety levels and good tolerability, thus, warranting further investigation [24]. However, combination therapy with both antifibrotics is not a standard treatment for IPF at the moment. Thus, it is important to treat IPF patients by individually administering these two antifibrotics in current practice. Theoretically, if one antifibrotic does not work or cannot be used due to ADRs, switching to an antifibrotic with a different mechanism of action may be clinically meaningful. Unfortunately, no studies have explored these issues to our knowledge. The present study focused on switching the antifibrotics in the treatment of IPF. In this retrospective study, 37 (14.1%) out of 262 IPF patients who had received antifibrotics eventually switched drugs during the observation period after a median period of 25.8 (12.7–35.3) months, which suggests that switching is not uncommon in real-world practice.

The most common reason for the switch was disease progression (45.9%), followed by GI side effects (32.4%) as determined by physicians. Compared to patients in the Switch-IPF group who switched for other reasons (Switch-IPF due to non-PD), the patients who switched due to disease progression (Switch-IPF due to PD) had significantly longer exposure periods to the first-line antifibrotics as well as shorter intervals from the end of first-line therapy to the start of second-line therapy. This is likely because the Switch-IPF due to non-PD patients mostly discontinued first-line antifibrotics due to ADRs within a short exposure periods from the initiation of first-line antifibrotics. Regarding the feasibility of second-line antifibrotics, among the 37 Switch-IPF patients, only eight (21.6%) discontinued second-line antifibrotics. None of the Switch-IPF due to PD patients had severe ADRs that caused them to discontinue second-line antifibrotics. By contrast, eight out of 20 Switch-IPF due to non-PD patients discontinued second-line antifibrotics because of ADRs. In particular, three patients terminated both first and second-line antifibrotics due to GI side effects. These issues are consistent with chemotherapy-induced nausea and vomiting (CNVI). In the field of oncology, experienced CNVI once was identified as a futured risk factor of CNVI in further chemotherapy [25]. These data suggest that patients who experience GI events during first-line antifibrotic therapy should be monitored for adverse GI effects during second-line antifibrotic therapy. Furthermore, treatment with intense prophylactic medicine for the GI side effects should be considered for these patients. Together, these results suggest that second-line antifibrotics are generally feasible, especially among patients that switched due to PD.

Although the causes of death did not significantly differ between Switch-IPF and Non-Switch-IPF patients, the survival times of Switch-IPF patients were significantly longer than those of Non-Switch-IPF patients. However, the baseline characteristics of Switch-IPF patients were not identical to those of Non-Switch-IPF patients. For instance, Switch-IPF patients had preserved lung function, lower incidences of AE history, and lower frequencies of LTOT usage compared with Non-Switch-IPF patients. Thus, we employed propensity score-matched analysis and multivariate Cox proportional analysis to adjust for these baseline differences. The propensity analysis adjusted by age, sex, FVC (%), AE history, and LTOT usages revealed that Switch-IPF patients still demonstrated significantly longer survival times than Non-Switch-IPF patients. Furthermore, the multivariate Cox proportional analysis revealed that patients with IPF who switched antifibrotics were identified as a population with better prognoses. Because 48 Non-Switch-IPF patients (21.3%) discontinued antifibrotic treatment during the observation period, we compared the prognoses between the Switch-IPF patients with Non-Switch-IPF Discontinued cases and Non-Switch-IPF Discontinued cases, respectively. We found that Switch-IPF patients still demonstrated significantly longer survival times than Non-Switch-IPF Discontinued cases and Non-Switch-IPF Discontinued cases. Collectively, these results suggest that switching antifibrotics from one to another may improve the prognosis among patients discontinuing first-line antifibrotics due to ADRs or those showing disease progression despite treatment.

The rationale for the longer survival times of the Switch-IPF patients compared to the Non-Switch IPF patients is not entirely clear. In the time-dependent Cox proportional analysis, “switching antifibrotics” itself did not show great impact to change prognosis of IPF, but efficacies of second-line therapy was not inferior to those of first-line therapy by the multistate model analyses (Fig. 5), suggesting importance of second-line antifibrotic therapy in patients with IPF. In the present study, approximately half of patients switched due to PD, whereas rest were due to ADRs. Additionally, patients with “Non-Switch IPF Discontinued cases” terminated antifibrotic therapy due to ADRs. When comparing the Switch-IPF patients and Non-Switch-IPF patients that discontinued antifibrotic treatment due to ADRs, it is reasonable to assume that the continuation of antifibrotic therapy can be meaningful even when the drug regimen changes. However, Switch-IPF patients still had significantly longer survival times compared with Non-Switch-IPF patients taking antifibrotics throughout the observation period. This finding suggests the possibility that sequential administration of two antifibrotics with distinct mechanisms of action may provide clinical benefits, especially if the first-line antifibrotics are not effective enough, which is similar to cancer therapy. There were also possibilities that impact of second-line antifibrotic therapy might be different by the reasons for switching antifibrotics; disease progression or ADRs. Although the small numbers of the present study did not allow further analyses, we believe that future prospective studies will elucidate these issues.

The present study had several limitations. Although the present study showed that patients with IPF who switched antifibrotics as a population with better prognosis, the prognostic implications of switching the antifibrotics were not completely clarified even evaluated using time dependent Cox proportional model and multistate model. Second, this study was retrospective and the sample size was not large enough to provide conclusive results. Furthermore, because pirfenidone was approved before nintedanib, most of the patients switched from pirfenidone to nintedanib rather than the other way around. The disease behavior, such as a decline in FVC (%) before and after antifibrotic therapy, was not evaluated. Additionally, the decision to switching antifibrotics either due to disease progression or ADRs is subjective, and there were no formulated protocols used by physicians. Collectively, these limitations may cause potential biases in this study. However, this study may provide novel clue about the sequential usage of two antifibrotics. These findings are important because only two antifibrotics are currently available, and neither drug completely halts disease progression. Thus, balanced and large-scale prospective studies are required to overcome these limitations.

In conclusion, the present retrospective study showed that 37 out of the 262 IPF patients who had received antifibrotics switched drugs during the observation period, and the second-line antifibrotics were generally well-tolerated. Moreover, patients with antifibrotics were identified as a population with better clinical outcomes as assessed by propensity-matched and multivariate Cox proportional analyses. Additionally, the present study showed survival benefit of second-line antifibrotic therapy was not inferior to that of first-line therapy Despite several limitations, the results of our study suggest that switching antifibrotics may be beneficial for the clinical management of IPF, especially among IPF patients discontinuing first-line antifibrotics due to ADRs or disease progression.

## Supplementary Information

**Additional file 1: Figure S1.** Survivals of patients with IPF with or without switching antifibrotics stratified by GAP system: Switched cases vs. Non-switched cases. Survival of patients with IPF GAP stage I with or without antifibrotic switching (**A**). Survival of patients with IPF GAP stage I with or without antifibrotic switching (**B**). *p* values were determined by the log-rank test. Cumulative survival probabilities from initiation of antifibrotic therapy were calculated. An event was defined as death from any cause and patients who were lost or still alive were censored at the last follow-up date.

**Additional file 2: Figure S2.** Survival of patients with IPF switching antifibrotics as analyzed by antifibrotic medication or cause of switching. Survival of patients with IPF switching antifibrotics by antifibrotic medication (**A**), and causes of switching (**B**). *p* values were determined by the log-rank test. Cumulative survival probabilities from initiation of antifibrotic therapy were calculated. An event was defined as death from any cause and patients who were lost or still alive were censored at the last follow-up date.

**Additional file 3:** **Supplementary Table 1.** Reasons for discontinuation of antifibrotics in 48 patients with Non-Switched IPF. **Supplementary Table 2.** Timeline of switching antifibrotics. **Supplementary Table 3.** Differences between the clinical characteristics in first-line and second-line anti-fibrotic therapy. **Supplementary Table 4.** Propensity-matched 74 patients with IPF with or without switches of the antifibrotics.

## Data Availability

The data that support the findings of this study are available from the corresponding authors upon reasonable request.

## Abbreviations

IPF: Idiopathic pulmonary fibrosis
ILDs: Interstitial lung diseases
FVC: Forced vital capacity
ADR: Adverse drug reaction
LTOT: Long term oxygen therapy
GI: Gastrointestinal
AE: Acute exacerbation
PD: Disease progression
BMI: Body mass index
CNVI: Chemotherapy induced nausea and vomiting
