# Supplementary figures and images for: Switching antifibrotics in patients with idiopathic pulmonary fibrosis: a multi-center retrospective cohort study
Source: BMC Pulm Med. 2021 Jul 12;21:221. doi: 10.1186/s12890-021-01587-3 (PMC8274040; doi:10.1186/s12890-021-01587-3)

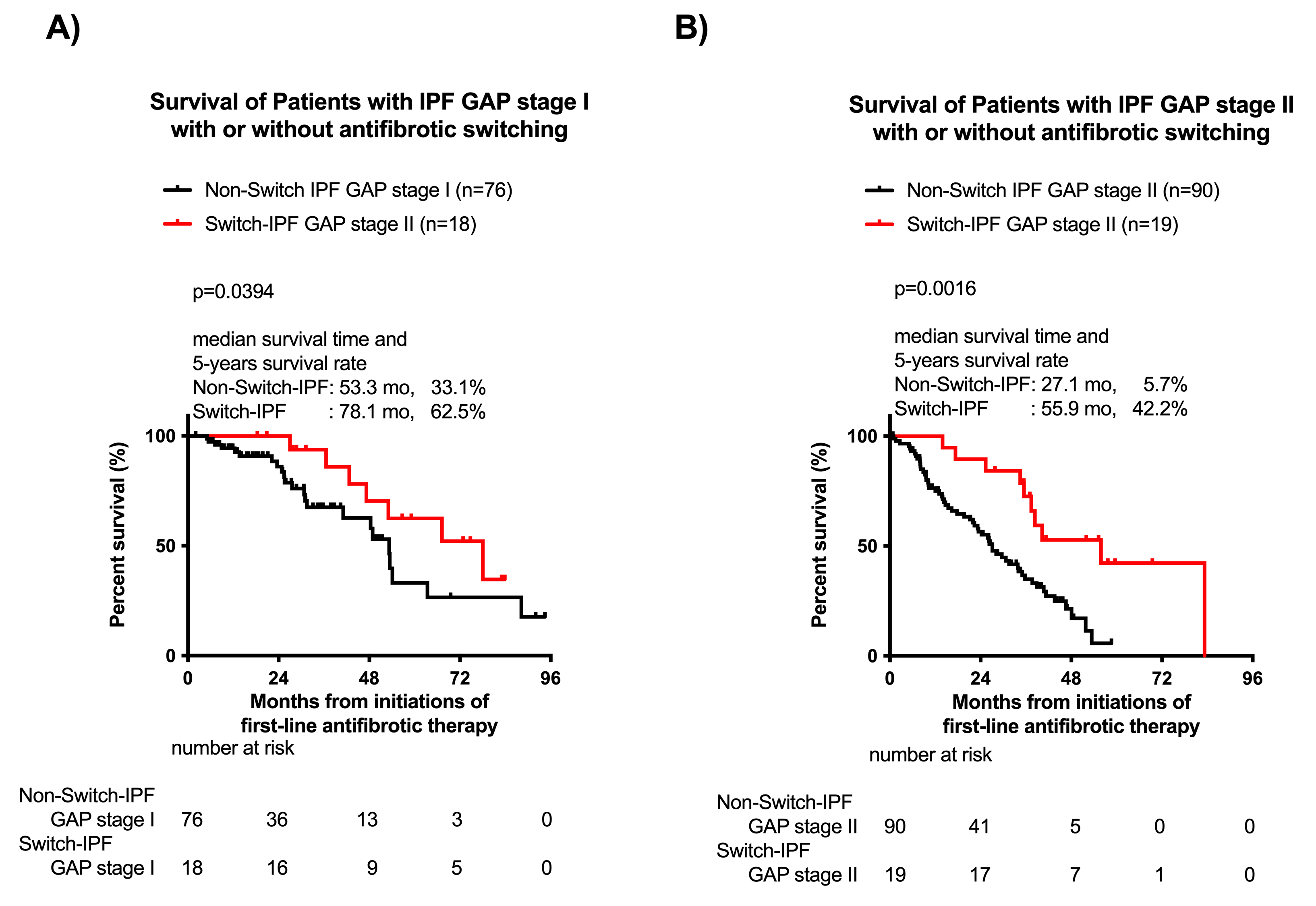

Supplement: Supplementary file 1 — Additional file 1: Figure S1. Survivals of patients with IPF with or without switching antifibrotics stratified by GAP system: Switched cases vs. Non-switched cases. Survival of patients with IPF GAP stage I with or without antifibrotic switching (A). Survival of patients with IPF GAP stage I with or without antifibrotic switching (B). p values were determined by the log-rank test. Cumulative survival probabilities from initiation of antifibrotic therapy were calculated. An event was defined as death from any cause and patients who were lost or still alive were censored at the last follow-up date. [file 12890_2021_1587_MOESM1_ESM.tiff]

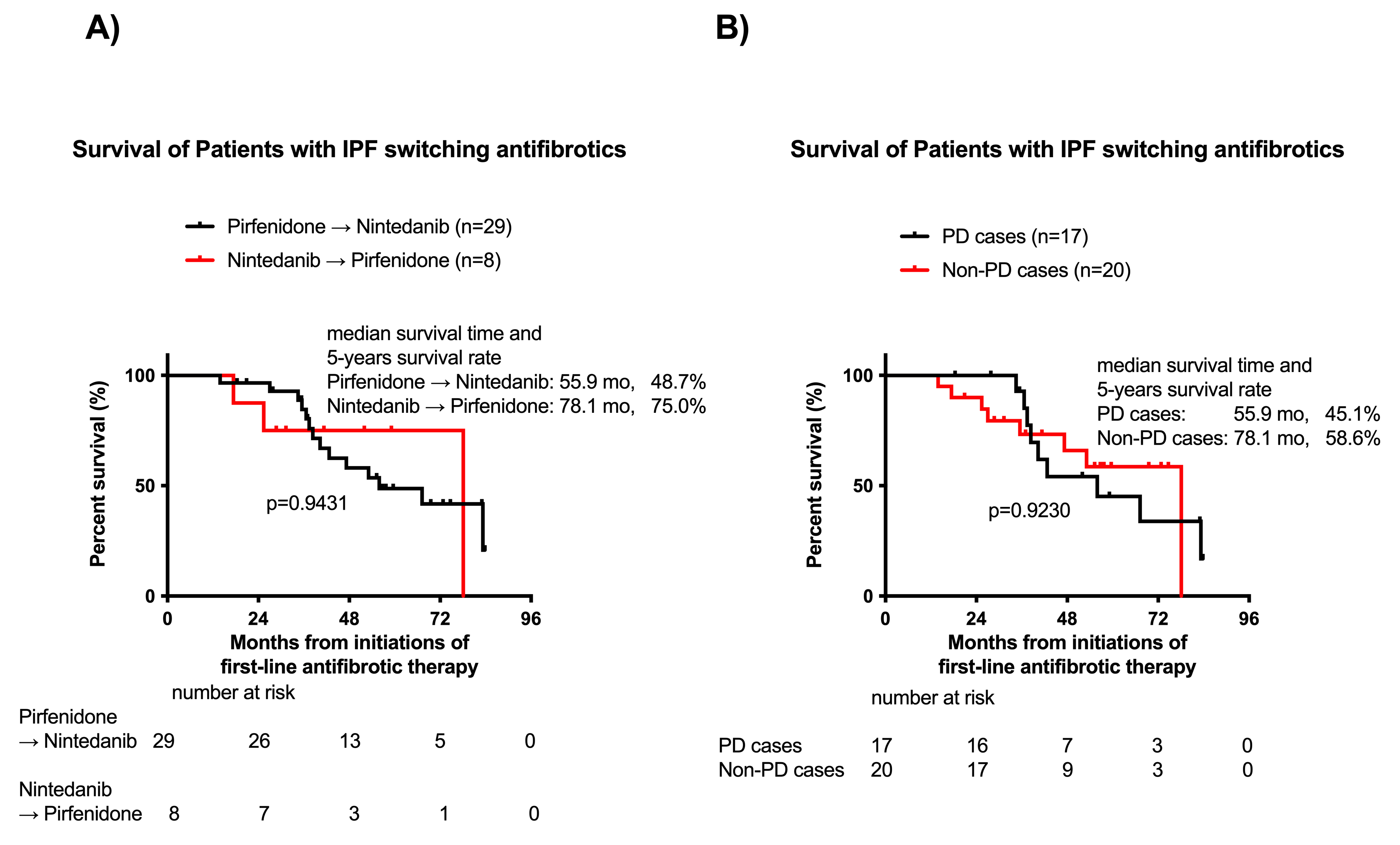

Supplement: Supplementary file 2 — Additional file 2: Figure S2. Survival of patients with IPF switching antifibrotics as analyzed by antifibrotic medication or cause of switching. Survival of patients with IPF switching antifibrotics by antifibrotic medication (A), and causes of switching (B). p values were determined by the log-rank test. Cumulative survival probabilities from initiation of antifibrotic therapy were calculated. An event was defined as death from any cause and patients who were lost or still alive were censored at the last follow-up date. [file 12890_2021_1587_MOESM2_ESM.tiff]
