# Supplementary material for: Switching antifibrotics in patients with idiopathic pulmonary fibrosis: a multi-center retrospective cohort study
Source: BMC Pulm Med. 2021 Jul 12;21:221. doi: 10.1186/s12890-021-01587-3 (PMC8274040; doi:10.1186/s12890-021-01587-3)
Supplement: Supplementary file 3 — Additional file 3: Supplementary Table 1. Reasons for discontinuation of antifibrotics in 48 patients with Non-Switched IPF. Supplementary Table 2. Timeline of switching antifibrotics. Supplementary Table 3. Differences between the clinical characteristics in first-line and second-line anti-fibrotic therapy. Supplementary Table 4. Propensity-matched 74 patients with IPF with or without switches of the antifibrotics. [file 12890_2021_1587_MOESM3_ESM.docx]

# Switching Antifibrotics in Patients with Idiopathic Pulmonary Fibrosis: A Multi-Center Retrospective Cohort Study

Yuzo Suzuki, M.D., Ph.D.^1^, Kazutaka Mori, M.D., Ph.D.^2^, Yuya Aono, M.D.^1^, Masato Kono, M.D., Ph.D.^3^, Hirotsugu Hasegawa, M.D., Ph.D.^4^, Koshi Yokomura, M.D., Ph.D.^4^, Hyogo Naoi, M.D.^1^, Hironao Hozumi, M.D., Ph.D.^1^, Masato Karayama, M.D., Ph.D.^1^, Kazuki Furuhashi, M.D., Ph.D.^1^, Noriyuki Enomoto, M.D., Ph.D.^1^, Tomoyuki Fujisawa, M.D., Ph.D.^1^, Yutaro Nakamura, M.D., Ph.D.^1^, Naoki Inui, M.D., Ph.D.^1^, Hidenori Nakamura, M.D., Ph.D. ^3^, Takafumi Suda, M.D., Ph.D.^1^

^1^Second Division, Department of Internal Medicine, Hamamatsu University School of Medicine, Hamamatsu, Japan

^2^Department of Respiratory Medicine, Shizuoka City Shimizu Hospital, Hamamatsu, Japan

^3^Department of Respiratory Medicine, Seirei Hamamatsu General Hospital, Hamamatsu, Japan

^4^Department of Respiratory Medicine, Seirei Mikatahara General Hospital, Hamamatsu, Japan

**Corresponding author:** Yuzo Suzuki, M.D., Ph.D.

**Address:** 1-20-1 Handayama Higashi-ku, Hamamatsu, Shizuoka 431-3192 Japan

**Tel:** +81-53-435-2263

**Fax:** +81-53-435-2354

**E-mail:** yuzosuzu@hama-med.ac.jp

**Running title:** Switch of antifibrotics in patients with IPF

**Conflicts of interest**: The authors declare that they have no competing interests.

***Supplementary Table 1. Reasons for discontinuation of antifibrotics in 48 patients with Non-Switched IPF***

| First-line treatment (n = 48)  [Pirfenidone (n = 28), Nintedanib (n = 20)] | |
| --- | --- |
| **Reasons**  Gastrointestinal disorders | 25 (52.1%), [14, 11] |
| Liver enzyme elevation | 6 (12.5%), [0, 6] |
| Disease progression | 5 (10.4%), [5, 0] |
| Photosensitivity | 1 (2.1%), [1, 0] |
| Rashes | 1 (2.1%), [1, 0] |
| Rashes and liver enzyme elevation | 1 (2.1%), [1, 0] |
| Gastrointestinal disorders and photosensitivity | 1 (2.1%), [1, 0] |
| Infection | 1 (2.1%), [1, 0] |
| Pneumothorax | 1 (2.1%), [0, 1] |
| Visual impairment | 1 (2.1%), [1, 0] |
| Patients’ will | 1 (2.1%), [1, 0] |
| Others | 4 (8.3%), [4, 0] |

***Supplementary Table 2. Timeline of switching antifibrotics***

|  | All patients  (n = 37) | PD patients*  (n = 17) | Non-PD patients**  (n = 20) | Non-PD cases vs. PD cases: *p*-value |
| --- | --- | --- | --- | --- |
| Duration between the initiation of first-line therapy and second-line therapy (months) | 25.8 [12.7–35.3] | 29.9 [24.2–41.6] | 16.9 [8.3–31.3] | 0.0412 |
| Exposure period for first-line antifibrotics (months) | 17.5 [5.0–31.2] | 29.9 [24.2–41.6] | 5.7 [2.8–13.2] | <0.0001 |
| Duration between the end of first-line therapy and the initiation of second-line therapy (months) | 0 [0–7.0] | 0 [0–0] | 5.3 [0–15.8] | <0.0001 |
| Observation period between the initiation of second-line therapy and censored (months) | 12.3 [8.1–30.3] | 11.6 [7.3–26.8] | 15.1 [8.7–38.7] | 0.5026 |
| Duration of exposure of the patients to second-line antifibrotics (months) | 11.6 [6.4–27.2] | 11.6 [7.3–26.8] | 11.7 [2.2–27.4] | 0.6258 |

*：PD patients; patients with IPF switched the antifibrotics due to progression of IPF

**: Non-PD patients; patients with IPF switched the antifibrotics due to reasons other than disease progression

***Supplementary Table 3. Differences between the clinical characteristics in first-line and second-line anti-fibrotic therapy***

|  | Switch-IPF cohort  First-line treatment  (n = 37) | Switch-IPF cohort  Second-line treatment  (n = 37) | *p*-value |
| --- | --- | --- | --- |
| Age, yr | 70.0 [65.5–74.0] | 73.0 [68.5–76.5] | 0.1326 |
| Sex, male/female | 31 (83.8%) / 6 (16.2%) | 31 (83.8%) / 6 (16.2%) | - |
| Pirfenidone / Nintedanib | 29 (78.4%), 8 (21.6%) | 8 (21.6%) / 29 (78.4%) | - |
| BMI, kg/m^2^ | 24.3 [21.3–25.3] | 21.8 [19.9–24.5] | 0.0386 |
| **Pulmonary Function Test** |  |  |  |
| FVC, %-pred | 73.2 [62.4–83.7] | 56.8 [49.5–76.3] (n = 35) | 0.0028 |
| FEV_1_, %-pred | 75.3 [67.6–87.6] | 64.5 [56.6–82.4] (n = 35) | 0.0253 |
| FEV_1_/FVC, % | 83.4 [79.9–88.9] | 88.1 [82.5–92.9] (n = 35) | 0.0264 |
| DLCO, % | 62.2 [50.2–67.6] (n = 36) | 48.8 [38.2–60.9] (n = 20) | 0.0133 |
| **Laboratory** |  |  |  |
| Hb, g/dl | 13.9 [13.2–15.5] | 13.6 [12.6–14.7] | 0.1927 |
| TP, g/dl | 7.6 [7.2–7.9] | 7.3 [6.9–7.5] | 0.0407 |
| Alb, g/dl | 4.1 [4.0–4.3] | 3.7 [3.5–4.1] | 0.0025 |
| LDH, U/L | 244 [209–272] | 237 [195–262] | 0.5579 |
| CRP, mg/dl | 0.2 [0.1–0.6] | 0.2 [0.1–0.8] | 0.5030 |
| KL-6, U/ml | 1124 [776–1473] | 1032 [696–1731] | 0.9736 |
| SP-D, ng/ml | 232 [136345] | 241 [154–377] | 0.7740 |
| **Treatment** |  |  |  |
| None | 30 (81.1%) | 17 (45.9%) | 0.0034 |
| LTOT | 6 (16.2%) | 21 (56.8%) | 0.0006 |
| Immunosuppressants | 3 (8.1%) | 5 (13.5%) | 0.7106 |
| Prednisolone | 3 | 5 | - |

BMI; body mass index, FVC; forced vital capacity, FEV_1.0_; forced expiratory volume in 1.0 second, DLCO; diffuse capacity of the lung for carbon monoxide, KL-6; Krebs von den Lunge-6, SP-D; surfactant protein-D, LTOT; long-term oxygen therapy

***Supplementary Table 4. Propensity-matched 74 patients with IPF with or without switches of the antifibrotics***

|  | Non-Switch-IPF cohort (n = 37) | Switch-IPF cohort  (n = 37) | *p*-value |
| --- | --- | --- | --- |
| Age, yr | 71.0 [67.5–74.0] | 70.0 [65.5–74.0] | 0.8878 |
| Sex, male/female | 32 (86.5%) / 5 (13.5%) | 31 (83.8%) / 6 (16.2%) | 1.0000 |
| cIPF / UIP/IPF | 29 (78.4%) / 8 (21.6%) | 31 (83.8%) / 6 (16.2%) | 0.7676 |
| Diagnosis to antifibrotic therapy, months | 12.1 [3.3–47.1] | 13.6 [1.7–31.5] | 0.4111 |
| First-line treatment: Pirfenidone / Nintedanib | 22 (59.5%), 15 (40.5%) | 29 (78.4%), 8 (21.6%) | 0.1309 |
| Exposure period for first-line antifibrotics, months | 22.0 [9.9–39.5] | 17.5 [5.0–31.7] | 0.2474 |
| History of acute exacerbation | 0 | 0 | - |
| Never / former & current smoker | 4 (10.8%), 33 (89.2%) | 6 (16.2%), 31 (83.8%) | 1.000 |
| Smoking pack-year | 30.0 [7.0–43.0] | 40.0 [18.0–59.0] | 0.1586 |
| BMI, kg/m^2^ | 23.0 [20.8–26.5] | 24.3 [21.3–25.3] | 0.8499 |
| **Pulmonary Function Test** |  |  |  |
| FVC, %-pred | 73.5 [63.0–81.3] | 73.2 [62.4–83.7] | 09698 |
| FEV_1_, %-pred | 74.1 [68.1–93.0] | 75.3 [67.6–87.6] | 0.6615 |
| FEV_1_/FVC, % | 85.4 [79.7–89.6] | 83.4 [79.9–88.9] | 0.3961 |
| DLCO, % | 64.1 [43.4–77.6] (n = 35) | 62.2 [50.2–67.6] (n = 36) | 0.8811 |
| **GAP stage, I, II, III** | 17 (47.2%), 17 (47.2%), 2 (5.6%) | 18 (48.6%), 19 (51.4%), 0 (0%) | 0.3454 |
|  |  |  |  |
| **6-minute walk test** |  |  |  |
| Distances, m | 435 [371–519] (n = 28) | 432 [345–515] (n = 28) | 0.7940 |
| Minimum SpO_2_ < 90% | 21/28 (75.0%) | 19/28 (67.9%) | 0.7681 |
| **UCG** |  |  |  |
| TRV ≥ 2.9 m/s | 2 (8.0%) (n = 25) | 2 (8.7%) (n = 29) | 1.0000 |
| **Laboratory** |  |  |  |
| Hb, g/dl | 13.7 [12.8–14.9] | 13.9 [13.2–15.5] | 0.2967 |
| TP, g/dl | 7.6 [7.1–7.9] | 7.6 [7.2–7.9] | 0.7774 |
| Alb, g/dl | 4.1 [3.8–4.2] | 4.1 [4.0–4.3] | 0.4167 |
| LDH, U/L | 216 [196–249] | 244 [209–272] | 0.0646 |
| CRP, mg/dl | 0.2 [0.1–0.6] | 0.2 [0.1–0.6] | 0.5180 |
| KL-6, U/ml | 896 [729–1380] | 1124 [776–1473] | 0.4079 |
| SP-D ng/ml | 212 [139–373] | 232 [136–345] | 0.956 |
| **Treatment** |  |  |  |
| None | 31 (83.8%) | 30 (81.1%) | 1.000 |
| LTOT | 6 (16.2%) | 6 (16.2%) | 1.000 |
| Flow rate during rest |  |  |  |
| (< 1, 1–3, or > 3 L/min) | 3, 3, 0 | 4, 2, 0 |  |
| Immunosuppressants | 2 (5.4%) | 3 (8.1%) | 1.000 |
| PSL | 1 | 3 |  |
| PSL + Tac | 1 | 0 |  |

BMI; body mass index, FVC; forced vital capacity, FEV_1.0_; forced expiratory volume in 1.0 second, DLCO; diffuse capacity of the lung for carbon monoxide, GAP; Gender-Age-Physiology, UCG; ultrasound echocardiogram, TRV; Tricuspid regurgitant jet velocity, KL-6; Krebs von den Lunge-6, SP-D; surfactant protein-D, LTOT; long-term oxygen therapy, PSL; prednisolone, Tac; tacrolimus
